# Regular measurement is essential but insufficient to improve quality of healthcare

**DOI:** 10.1136/bmj-2022-073412

**Published:** 2023-03-13

**Authors:** Ambrose Agweyu, Kathleen Hill, Theresa Diaz, Debra Jackson, Binyam G Hailu, Moise Muzigaba

**Affiliations:** 1 Department of Epidemiology and Demography KEMRI-Wellcome Trust Research Programme, Nairobi, Kenya; 2 Department of Infectious Disease Epidemiology, London School of Hygiene and Tropical Medicine, London, UK; 3 Jhpiego, Baltimore, Maryland 21231, USA; 4 Department of Maternal Newborn, Child, and Adolescent Health, and Ageing, World Health Organization, Avenue Appia 20, 1202 Geneva, Switzerland; 5 School of Public Health, University of the Western Cape, Bellville, South Africa; 6 Data and Analytics Section, Unicef, New York, USA; 7 World Health Organization Country Office, Free Town, Sierra Leone

## Abstract

**Ambrose Agweyu and colleagues** argue that large scale improvements in quality of healthcare require strong change management as well as health information systems that can provide continuous and rapid feedback

Evidence on the detrimental effect of low quality health systems on preventable mortality worldwide has accelerated investments in large scale healthcare improvement.^1^ Regular measurement of quality of care is a core principle of quality improvement programmes that has been promoted in some low resourced settings as the primary means to improve quality of healthcare— that is, the degree to which health services for individuals and populations are effective, safe, and people centred.^2^

Advances in information technology over the past decades, along with a growing demand for accountability and regular measurement of quality of care, have resulted in a proliferation of indicators, tools, and approaches to measuring the performance of health systems.^3^ ^4^ Routine health information systems (RHIS) that capture high quality data to facilitate regular use of data to monitor realtime trends in healthcare processes and outcomes are essential.

Regular measurement, however, will not improve healthcare on its own. Measurement must be coupled with specific actions to improve care, including change management processes to achieve and sustain large scale healthcare improvements. This realignment requires an appreciation of the challenges associated with the current model in healthcare, especially in low resource settings.

## Unlocking potential of routine health information systems

High quality health information on which to base decisions is needed by patients and their communities, healthcare providers and managers, insurers and other payers, governments, and international development agencies. Since health information systems in many settings with high mortality have been developed, to some extent, to facilitate the aggregation of information at subnational, national, and global levels to fulfil reporting requirements, they are often not fully used to improve health service management and quality at the local level. Faced with the challenge of satisfying the diverse information needs of different consumers, health systems often struggle with data generation, collation, analysis, and reporting, let alone using these data to establish or monitor quality improvement efforts.

Health information systems in countries across all income levels fall short of their potential to contribute to improving health system performance because of three interrelated factors: technical limitations, including poorly structured data collection tools, limited interoperability, and inadequate investment in maintenance, support, and data privacy^5^ ^6^ ^7^; behavioural factors, such as poor motivation to generate high quality data^8^ ^9^; and organisational determinants often arising from weak governance and resource constraints resulting in understaffing, commodity shortages, and limited workforce skills in data management and data use for quality improvement.^9^ ^10^ Further, in many settings, health information technologies do not capture important measures of quality of care regularly.

In many low resource settings, data on quality of care in health information systems (eg, health facility registers, patient records) are often limited and may be inconsistently documented in paper based records. Local information on quality of care therefore depends on infrequent cross-sectional household and health facility surveys. Surveys can generate important information on quality of care and fill important gaps in reporting the state of health systems where critical data are otherwise unreliable or unavailable. In practice, however, surveys are conducted only every 5-10 years in many countries and are both labour intensive and costly. These surveys are also funded and coordinated by external organisations at the expense of sustainable country capacity, usually consist of a restricted sample of households or facilities, and, critically, are rarely directly fed back into the improvement of routine services. In short, these assessments are too infrequent to provide usable information to inform actions in real time to improve health system performance.

Currently, people working in measurement, technical specialties (eg, a particular health issue or disease), and quality improvement in both high and low income settings often operate in siloes and do not have the skills or support to enable them to collaborate effectively to unlock the potential of routine health information systems to inform quality improvement efforts. For example, in many countries, health information, technical, and quality improvement divisions in the ministry of health operate relatively independently and sometimes even in competition. Similarly, regional health information officers, technical programme managers, and quality of care directors may focus on their respective domains without the training, direction, rationale, or support to contribute their respective expertise to collaborative efforts. 

For example, unless a district child health programme manager, information officer, and quality of care focal point work together closely to support collection and regular analysis of trends in child health quality of care indicators, they are unlikely to implement coordinated actions to improve care of children and will not know if their actions are associated with improved quality of care. In many settings, however, technical managers focus on technical guidelines, training, and supervision, while information officers focus on reporting child health indicators into a national health information system, missing the opportunity for coordinated action.

Global quality improvement efforts and guidance often reinforce these siloes. For example, global recommendations on quality of care indicators often fail to consider how suggested indicators might be feasibly measured, let alone whether they will be useful to those leading health system improvement efforts in different settings. For example, a recommendation for an indicator to monitor percentage of children with anaemia who were treated according to WHO guidelines may only be feasible in contexts where haemoglobin testing is performed routinely for all patients.

In most highly resourced settings, routine health information systems have evolved to provide detailed longitudinal data at the individual patient level. For example, web based health record systems accessible to patients and healthcare providers allow providers and people with diabetes to monitor trends in blood glucose levels, an important quality outcome measure of diabetes care.^11^ The widespread rollout of electronic health records with integrated decision support and data quality assurance has the potential to promote care continuity, augment evidence based clinical decision making, and enhance opportunities for comparison and learning across settings.^12^ However, effective implementation of electronic records is costly and often inaccessible for lower income settings.^13^

In recent years, many countries with less robust health information systems have incorporated quality of care data elements into their routine health information systems.^14^ Sierra Leone, one of several countries in a network to improve quality of maternal, newborn, and child healthcare, has created a maternal and newborn health quality of care module in their national information system to enable the efficient capture, use, and reporting of a set of core network indicators generated from data in health facility registers (eg, pre-discharge neonatal mortality). The healthcare quality indicators prioritised are tailored to the specific needs of those working at different levels of the health system, including national policy makers and leaders, regional and district managers, and healthcare facility quality improvement teams.^15^

## Measurement must be linked to change management

Irrespective of country resources and how data are collected, measurement of quality of care indicators needs to be accompanied by strong leadership for change if it is to translate into improved patient outcomes. Change management, as we are using it here, refers to leadership within an organisation or health system for designing, testing, and implementing changes to close gaps in quality of care using realtime measurement.^16^ Changes are focused on the root causes of quality of care problems in a local setting, identified through systematic problem solving methods. Root causes of poor quality may span multiple health system areas such as health workforce (eg, weak leadership, skills, motivation); organisation of care (eg, inefficient care flow in a clinic); governance and policies; and infrastructure and commodities among others. 

Consider a local health team that wants to take action to improve early detection and management of high blood pressure in pregnant women in a setting with a high prevalence of hypertensive disorders in pregnancy. The team will need to identify, agree, and monitor a small number of process and outcome measures to know whether the changes they introduce (such as assigning an auxiliary health worker to measure women’s blood pressure before being seen by a midwife in a busy clinic) are improving care. These might include the percentage of women with a blood pressure check at every antenatal visit and the percentage of pregnant women being treated for hypertension who have a normal blood pressure value.

Without regular measurement, managers and facility teams will not know whether the changes they are introducing are associated with improved care. Conversely, care will not improve unless specific actions are taken based on an understanding of the root causes of poor quality of care in a local setting (eg, a single midwife in a busy clinic does not have time to check the blood pressure of every pregnant woman). Both qualitative and quantitative data are important for evaluating and guiding improvement.^16^ Qualitative data from providers and health service users enable assessment of the feasibility, acceptability, and sustainability of changes being introduced to improve care in a local healthcare system. At the same time, care for pregnant women with hypertensive disorders of pregnancy is unlikely to improve or be sustained unless managers and their teams embrace the soft skills required to establish a culture that supports change management. 

Improvements in care are more likely to be both sustained and widespread if all facilities in a given catchment area regularly monitor and share their results and the changes they are making as part of an intentional learning system. Broader system improvement can occur if guided by strong leadership for change, if quality improvement efforts are embraced by managers, and if lower performing facilities can be identified and provided with additional support. In a systematic review of the effectiveness of strategies to improve the performance of healthcare providers in low and middle income countries, group problem solving (often in the context of multisite improvement collaboratives incorporating regular peer-to-peer learning) showed moderate to large improvements in provider practice outcomes.^17^ Peer-to-peer learning can be supported by using existing processes in a local health system, such as in-person or virtual learning meetings, continuing professional development activities, social media forums, and webinars.

One example of a successful multisite learning network focused on quality improvement is a critical care network of 42 intensive care units in nine Asian countries. Clinicians, researchers, and policy makers representing facilities in the network identified a lack of reliable data as a barrier to the implementation of local quality improvement activities. The network leadership mobilised grant funding to support the platform activities, convened network members to agree on a common set of clinical measures that could be captured consistently in near real time, and supported the continued use of the measures to drive improvement across the network.^18^ Multisite learning networks also support efficient locally driven observational, implementation and interventional research that has the potential to affect policy.^19^ ^20^ While such networks provide promising opportunities for implementing large scale change, they require considerable investment to establish and support, again reflecting that measurement needs to be linked to change management in order to generate sustainable quality improvement. 

Information about providers’ performance compared with quality standards through external or self-audit can be an effective motivator for change among health professionals. Audit and feedback generally leads to small but potentially important improvements in professional practice.^21^ A paediatrician led network in Kenya, for example, has shown substantial improvements in routine clinical practices, including ascertainment of HIV status, screening for malnutrition, and documentation of oxygen saturation in children across more than 20 hospitals in Kenya, through audit and feedback.^19^ ^22^ Although the effectiveness of feedback depends on several factors, including the baseline performance and profession of the recipient, a follow up analysis to a 2012 Cochrane review suggests feedback is most effective when delivered by a supervisor or respected colleague, presented frequently, and when it features both specific goals and action plans to facilitate change management.^23^

## Changing the status quo

Given the importance of quality of care for achieving global and country targets set out in the sustainable development goals (SDGs) and the gross inequities in SDG indicators within and across countries, more must be done to align real time measurement of quality of care and interventions to improve care. This entails improving the availability of information that can be used at local level rather than for administrative reporting. Priority areas include the adaptation of health information systems to capture structured data on quality of care across different levels of the health system without imposing too much extra burden on healthcare workers who generate the data and to facilitate regular use of these data to guide quality improvement.

Curriculums for training all types of health worker should include a greater range of quality improvement and measurement skills, and these could be taught in multidisciplinary groups to promote collaboration. Likewise, governance and learning systems should demand and facilitate close collaboration among technical, measurement, and quality of care leads and managers. This strengthened collaboration may also help overcome common gaps and biases in health system data, such as data manipulation, by promoting transparency and accountability among those responsible for data generation and use.^24^

Investments in strengthening data systems and the capacity of health workers to measure quality of care must equally prioritise the ability to understand the root causes of poor quality and to implement actions to address these causes. This requires a shift in thinking so that health information officers, clinicians, quality of care officers, managers, and other stakeholders collaborate effectively to regularly measure quality of care and draw on that data to inform change management as part of quality improvement. Without this alignment, measurement cannot be expected to improve care.

**Key messages**

- Regular measurement of quality of care is a core principle of improving healthcare
- Routine health information systems have the potential to measure quality of care in real time
- Measurement alone will not improve healthcare unless linked to change management and backed by strong leadership and learning systems
- Successful alignment of measurement with health system change demands close collaboration among health information officers, clinicians, quality of care officers, managers, and other stakeholders

## Notes

Contributors and sources: AA is a Kenyan paediatrician and a clinical epidemiologist who has published extensively on using routine data to improve care, study patient outcomes, and evaluate effectiveness of interventions. KH is a family physician with 20 years of service delivery, programme management, and quality improvement experience in low, middle, and high income settings. MM is a quality of care metrics and improvement science specialist with over 15 years of work experience in low and middle income countries. He co-chairs the WHO’s life course quality of care metrics technical working group with KH and DJ. DJ is deputy director of the MARCH Centre and has broad expertise in implementation research and quality of care measurement. TD has wide expertise in maternal, newborn, child, and adolescent health measurement. BGH has wide expertise in quality of care programming and measurement. AA was responsible for developing the initial draft of the manuscript and finalising it for submission. KH and MM were responsible for developing the original manuscript concept, coordinating the writing process, and providing technical and contextual input. DH, TD, and BGH reviewed and provided input to multiple drafts. The authors alone are responsible for the views expressed in this article and they do not necessarily represent the views, decisions, or policies of the institutions with which they are affiliated.

This article is part of a collection proposed by the World Health Organization and the World Bank and commissioned by *The BMJ*. *The BMJ* peer reviewed, edited, and made the decision to publish these articles. Article handling fees are funded by the Bill and Melinda Gates Foundation. Jennifer Rasanathan, Juan Franco, and Emma Veitch edited this collection for *The BMJ*. Regina Kamoga was the patient editor.
